# The Luohu Model: A Template for Integrated Urban Healthcare Systems in China

**DOI:** 10.5334/ijic.3955

**Published:** 2018-10-12

**Authors:** Xin Wang, Xizhuo Sun, Fangfang Gong, Yixiang Huang, Lijin Chen, Yong Zhang, Stephen Birch

**Affiliations:** 1 School of Public Health, Health Development Research Center, Sun Yat-sen University, 74 Zhongshan 2nd Road, Guangzhou, CN; 2 Shenzhen Luohu Hospital Group, No. 47 Youyi Road, Shenzhen, CN; 3 Centre for the Business and Economics of Health, University of Queensland, AU

**Keywords:** integrated care, hospital group, district healthcare system

## Abstract

**Introduction:**

Emerging from the epidemiological transition and accelerated aging process, China’s fragmentated healthcare systems struggle to meet the demands of the population. On Sept 1^st^ 2017, China’s National Health and Family Planning Commission encouraged all cities to learn from the Luohu model of integration adopted in Luohu as an approach to meeting these challenges. In this paper, we study the integration process, analyze the core mechanisms, and conduct preliminary evaluations of integrated policy development in the Luohu model.

**Policy development:**

The Luohu hospital group was established in Aug 2015, consists of five district hospitals, 23 community health stations and an institute of precision medicine. The group adopted a series of professional, organizational, system, functional and normative strategies for integrated care, which was provided for the residents of Luohu, especially for the elderly population and patients with chronic conditions. According to a preliminary evaluation of the past two years, the Luohu model showed improvement in the structure and process towards integrated care. New preventive programs conducted in the hospital group resulted in changes of disease incidence. Residents were more satisfied with the Luohu model. However, spending exceeded the global budget for health insurance because of short-term increases in the demand for health care.

**Lessons learned:**

First, engagement of multiple stakeholders is essential for the design and implementation of reform. Second, organizational integration is a prerequisite for integrated care in China. Third, effective care integration requires alignment with payment reforms. Fourth, normative integration could promote collaboration in an integrated healthcare system.

**Conclusion:**

Core strategies and mechanisms of the Luohu model will promote integrated care in urban China and other countries facing the same challenges. However, it is necessary to study the effects of the Luohu model over the long term and continue to strive for integrated care.

## Introduction

Healthcare systems worldwide have been designed primarily to deal with single, acute, and short-term illnesses [1,2]. However, emerging from the epidemiological transition and an accelerated population aging process, fragmentated healthcare provided by traditional healthcare systems in most countries cannot meet the demands of the population, especially the elderly, many of whom often have chronic diseases [3,4,5]. Moreover, the traditional healthcare system has suffered from low levels of efficiency, cost escalation, and poor patient experiences [6,7]. As a developing country getting old before getting rich, China’s healthcare systems are facing considerable challenges of fragmented care. In China, there were 231 million people aged 60 years or over in China (16.7% of the population) [8] in 2016. Among them, more than 100 million had at least one chronic noncommunicable disease [9]. Further, 626 deaths, 21,020 disability adjusted of life years (DALY) and 8,879 years lived with disability (YLDS) per 100,000 population were attributed to chronic noncommunicable disease [10]. It is predicted that the percentage of people aged 60 or over will increase from 12.4% in 2010 to 28% in 2040 [11]. These challenges indicate a need for urgency in transition from fragmented care to integrated care in China’s healthcare systems.

Current fragmented healthcare delivery in China is hospital-centered and treatment-dominated, with little effective collaboration among institutions in different tiers of the system [12]. In the 1980s, China moved to a market economy, and the government dramatically reduced hospital funding. Responding to these reductions, hospitals tried to earn revenues by providing more profitable health care, primarily diagnosis and treatment rather than prevention and rehabilitation. The traditional three-tier healthcare system collapsed, and primary health care stations no longer served as gatekeepers [13]. Since new health reforms were introduced in 2009, China’s government has been encouraging health care provision in primary health stations by financial subsidies and a program entitled Equalization of Basic Public Health Services [14]. However, measures to improve collaboration among institutions and reduce fragmentation of services have been insufficient.

Over the last decade, integrated care has been suggested as one strategy for promoting coordinated health care delivery, improving the quality of care and reducing costs [15,16,17]. In 2016, the report “Deepening health reform in China” [18], published by the World Health Organization (WHO), World Bank (WB), and the Chinese Government, proposed strengthening healthcare in China through a tiered health care delivery system in accordance with a People-Centered Integrated Care model. In April 2017, the General Office of the State Council issued a Guideline for constructing Medical Consortia [19]. In the guideline, four types of medical consortia were suggested: hospital groups in urban areas, medical associations in rural areas, cross-regional specialist alliances and tele-collaboration networks in remote areas. Medical consortia thus became a main means for achieving People-Centered Integrated Care. On Sept 1^st^ 2017, China’s National Health and Family Planning Commission introduced the Luohu model, an approach to healthcare integration pioneered in Luohu District, to the entire country and encouraged all cities to learn from it [20]. Subsequently, more than 1,500 policy makers from health and other social sectors in 321 cities received on-site training in the Luohu model.

The aims of this study are to introduce the Luohu model, to evaluate its effects and to explore lessons learned. With enhancement of the Belt and Road health collaboration [21,22], health reforms in urban China may have a considerable impact on other countries’ health systems, especially low- and-middle-income countries facing the same challenges. Moreover, features of healthcare integration in China, which may differ from those in European countries, may provide references for other countries.

## Development of the Luohu Model

### Background of Shenzhen City and the Luohu District

China’s Reform and Opening in 1980s began in Shenzhen City, which, in 2016, ranked first in economic competitiveness among cities nationwide. Luohu is one of the ten districts in Shenzhen, with an area of 78.36 km^2^ and a population of 1.4 million. Per capital Gross Domestic Product (GDP) in Luohu was $25,200 in 2016 [23]. It also has the largest proportion of the elderly residents in Shenzhen. It is estimated that the number of elderly individuals and patients with chronic conditions exceeds over 451,000. The government of Luohu has the tradition of reform, not only in economy but also in health.

Luohu District has five district-level hospitals and 83 community health stations. While most cities with two-level primary health care institutions [24], Luohu has only one-level community health stations. Furthermore, all 83 community health stations are affiliated with one of the five hospitals. For example, the community health management center in the Luohu General Hospital is in charge of 18 community health stations, managing human resources, finance, assets and service delivery in each station. Also located in Luohu is a city-level general hospital with 2,000 beds, under the charge of the Shenzhen city government. The numbers of beds and physicians in the city hospital exceed those in the five independent district hospitals. The expansion of the city hospital has been associated with the weakening of the ability of district hospitals and community health stations to provide care as patients are free to seek services at the city hospital directly. Therefore, “line up for 3 hours, treatment for 3 minutes” became a problem in Luohu, especially for the elderly and patients with chronic diseases. The Luohu health reform system aims to achieve “less illness, fewer hospital admissions, lower financial burdens, and better services” [25] by development of a community-based and prevention-oriented integrated care system.

### Process of the Luohu reform

**Figure 1:**
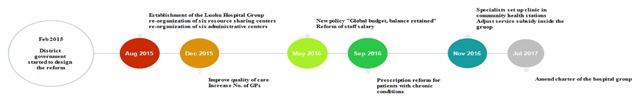
Timeline of the Luohu reform.

**Figure 2:**
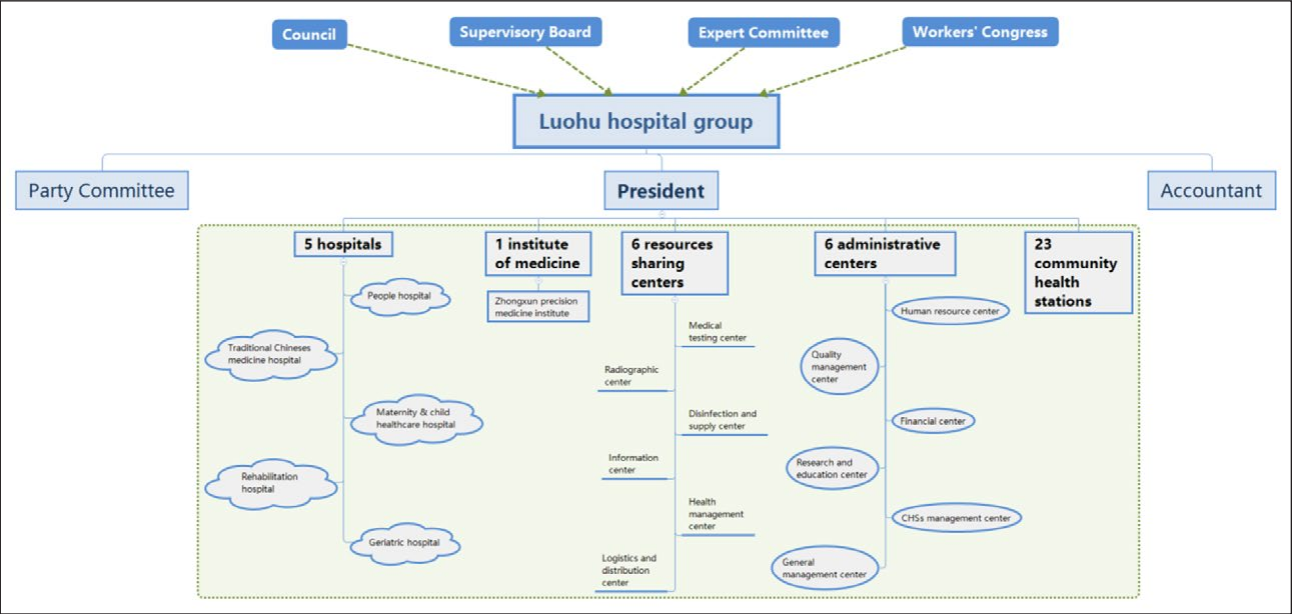
Organizational structure of the Luohu hospital group.

Figure 1 shows the timeline of the Luohu reform. In February 2015, the district government began with the concept “Shifting focus from treatment to health”. After 10 rounds of expert consultations, the Luohu hospital group was established in Aug 2015. It consists of five district hospitals, 23 community health stations, and an institute of precision medicine, along with six resource sharing centers and six administrative centers (Figure 2). After being established, the hospital group adopted a series of reforms. In Dec 2015, a Quality Management Center took action to improve the quality of care in all institutions, especially in community health stations. In May 2016, a new health insurance policy, “Global budget, balance retained”, was introduced which funded hospitals via global budgets and allowed institutions to retain any funds not spent during the financial year. At the same time, salary reform was instituted to motivate staff. In Sept 2016, a prescription of three month was allowed for patients with one of ten types of chronic conditions to seek treatment, to avoid unnecessary outpatient visits to district hospitals. In Nov 2016, the hospital group encouraged specialists in district hospitals to set up clinics in community health stations, to increase the proportion of first contacts occurring in primary health stations. In July 2017, the charter of the hospital group was amended based on lessons learned from the preceding two years. Thus, the establishment of the hospital group provided the basis for the ensuring reforms.

### Strategies of the Luohu model

**Figure 3:**
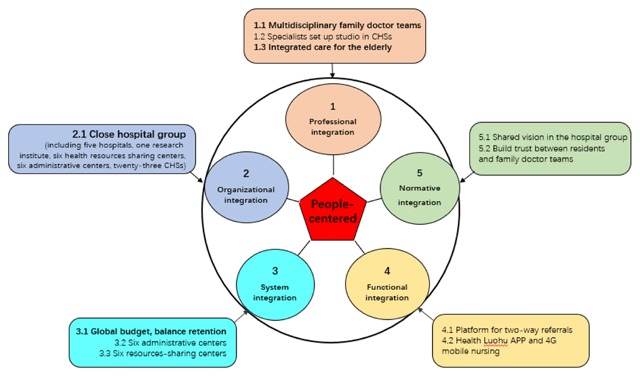
Strategies for an integrated care system in Luohu.

Based on the Rainbow Model of integrated care developed by Valentijn and colleagues [26,27], integration processes at the macro-level (system integration), meso-level (organizational and professional integration), micro-level (clinical integration) and cross-level (functional and normative integration) contribute to integrated care. The strategies taken in Luohu for constructing a community-based and prevention-oriented integrated care system are summarized in Figure 3. There are strategies regarding professional, organizational, system, functional and normative integration. Among these are three core strategies for establishment of the hospital group. In the hospital group. Detailed integrated care was provided for the residents, especially for the elderly and patients with chronic conditions.

#### Three core strategies

The first strategy was the establishment of a hospital group in the form of an independent corporation. Six resource-sharing centers were organized using the resources of the respective centers in the former five hospitals, including a medical testing center, a radiographic center, an information center, a health management center, a logistics and distribution center and a disinfection and supply center, along with six administrative centers to manage all institutions in the group, including a human resource center, a financial center, a quality management center, a research and education center, a community health station management center and an integrated management center. Twelve centers provide resources and management for the entire hospital group. After the organizational integration, there were 1,172 beds, 3,479 staff, and 778 physicians in the group. The president is responsible for the group under the leadership of a council. Among 12 council members, there are policy makers from the District Government, Health and Family Planning Commission and resident representatives. The president has the capacity to make plans and coordinate activities for all institutions in the group. All institutions in the group share management, services, benefits and responsibilities.

The second strategy was the development of a new health insurance policy, “Global budget, balance retained”. Shenzhen’s Social Insurance Fund Administration, which is affiliated with the Human Resources and Social Security Bureau, is responsible for collecting and managing the social insurance fund of the entire city. Coordinated by Shenzhen’s Health and Family Planning Commission, Luohu became the first pilot for this new health insurance policy. The global budget of the hospital group in 2016 was given by the total cost of health insurance for registered residents in the previous year multiplied by the average growth rate of the health insurance fund in 2016.

It should be noted that registered residents could seek health care in any institutions inside or outside the hospital group. Wherever the residents received health care, the hospital group had to pay for them, unlike a Health Maintenance Organizations model where the organization is only accountable for care provided within the organization. Any surplus at the end of the year is retained and can be used for staff bonuses. The incentive and restraint mechanisms formed by this policy aimed to change the behavior of district hospitals. On the one hand, hospitals have to pay more attention to helping community health stations provide prevention and case management. Only in this way can they reduce illnesses and the demand for hospitalization and hence reduce the health insurance cost of the group. On the other hand, hospitals will make efforts to improve the quality of health services and gain the residents’ trust to avoid patients seeking health services outside the group. The cost for registered patients seeking treatment in hospitals outside the group will be paid by the group. To avoid physicians’ reducing services to increase the balance of health insurance, the quality management center is responsible for supervising physicians’ practices.

The third strategy involved building family doctor teams in each community health station. Family doctor teams play an important role in promoting effective resource utilization, reducing costs and improving patient satisfaction [28]. However, a dearth of general practitioners hindered the development of family doctor teams in China [29]. The Luohu hospital group recruited general practitioners throughout the world, hired international general practitioner experts for on-site clinical training and encouraged position shifts for some specialists. In community health stations, each family doctor team consists of a general practitioner (leader), nurses, health promotion staff and a public health physician and may also include specialists, pharmacists, nutritionists, and psychologists. Specialists are provided with an incentive of $100 per day to set up clinics in the community health stations or serve as a member of a family doctor team in community health stations in their spare time. A list of ten identified tasks of the family doctor team (including health education, case management for pregnant women etc.) was widely publicized to make residents understand the team’s responsibilities. The family doctor team was expected to change the behavior of staff in community health stations and provide a platform for the transition from hospital-centered treatment-dominated care to community-based prevention-first care.

#### Integrated care for the elderly

In Aug 2014, a Rehabilitation Center for the elderly was introduced by the general district hospital in Luohu and began to explore integrated care for the elderly [30]. After the establishment of the Luohu hospital group, an integrated care system for the elderly was formed based on home care and community care supplemented by hospitalization.

***Home care***. Staff in community health stations provide nursing, rehabilitation, and palliative care for the disabled elderly by setting up beds in the patients’ homes. The District’s Ministry of Finance provides a subsidy for home care. ***Community care*** delivery varies between communities. First, through collaboration with community health stations, day care centers in the community provide drug management, rehabilitation and health education for the elderly. Second, day care centers affiliated with hospitals provide treatment, nursing, rehabilitation, and case management for the elderly using multi-profession teams. Third, social service centers inside each community health station provide integrated care for the elderly. ***Hospitalization care***. The geriatric hospital in the hospital group provides not only nursing and rehabilitation services but also diagnostic and treatment services for the elderly.

#### Integrated care for patients with chronic conditions

The Luohu hospital group explored integrated care and case management for patients with chronic diseases. Public health physicians were allocated to community health stations and became members of family doctor teams. Health care delivery for patients with chronic diseases changed from treatment-dominated to prevention-first based on three strategies.

First, the group paid more attention to ***preventive care***. For example, regular lectures were given in each community and a Healthy Luohu app was designed for improving residents’ health literacy. In cooperation with the government, the group helped to construct two jogging trails for residents, to cultivate exercise. Moreover, free pneumonia vaccinations were provided for those over 60 years of age in 2016.

Second, the group introduced screening programs for diseases with high morbidity and mortality, with particular focus on cancers. Screening programs for breast cancer, cervical cancer, lung cancer, liver cancer, and gastrointestinal cancer were introduced [24], to support ***early diagnosis and treatment***.

Third, physicians prepared ***individualized healthcare plans*** for patients and provided medical treatment and non-drug guidance regularly in collaboration with general practitioners. There is a Referral Gateway between general practitioners in community health stations and hospitals in the group. Whenever patients need the ***services of specialists***, physicians will refer them to one of the group’s hospitals and continue to follow up.

## Evaluation of the Luohu model

### Framework

Devers and colleagues [31] suggested that healthcare integration be evaluated in three dimensions: readiness of integration (structure), internal process of integration (process), and outcomes of integration (outcome). Selection of the second- and third-level indicators was based on a review of the literature, as well as on the aims of and programs in the Luohu model. In this study, we adopted six indicators to evaluate “structure”, eight indicators system to evaluate “process” and 12 indicators to evaluate “outcome” (Table 1).

**Table 1:** Evaluation results of the Luohu model.

First-level indicator	Second-level indicator	Third-level indicator	Jun 2014–Jun 2015		Jun 2015–Jun 2016	Jun 2016–Jun 2017

Structure	Area of CHS*	Average area of CHS* (m^2^)	410		749.5	903
	Assets of CHS*	Assets of equipment of all CHSs* (million, $)	2.76		3.50	4.09
	Human resources of CHS*	No. of general practitioners	89		147	194
		No. of physicians providing public health services	2		4	30
		No. of specialists setting up practices in CHSs*	0		0	49
		No. of family doctor teams	0		198	238
Process	Utilization of general practitioners	No. of residents registered with general practitioners	0		0.15	0.58
		Proportion of residents registered with general practitioners (%)	0		12.5	38.7
	Utilization of inpatients	No. of inpatients in the hospital group	3021		5434	7034
		Proportion of inpatients hospitalized in the hospital group compared with all registered inpatients (%)	15.3		19.3	21.9
	Utilization in CHS*	No. of outpatients served by CHSs (million)	0.795		2.02	2.25
		Percentage of CHSs’* patients of all patients in the group (%)	29.49		36.87	4260
	Two-way referral	No. of down-referral patients	0		4365	5647
		No. of up-referral patients	1442		3084	4937
Outcome	Case Management	No. of patients with diabetes under case management	5624		8210	10220
		Proportion of diabetes patients under case management (%)	66.88		67.23	69.54
		No. of patients with hypertension under case management	19667		22579	24662
		Proportion of hypertension patients under case management (%)	65.5		66.0	68.2
		No. of patients with severe mental illness under case management	1113		1402	1335
		Proportion of patients with severe mental illness under case management (%)	10.6		11.2	11.8
	Incidence and mortality	Incidence of infectious disease (1/100,000)	351.5		350.7	300.4
		Mortality rate under 5 (‰)	2.5		2.4	1.3
		New cases of pneumonia	734	**	511	283
		New cases of cancer	631	**	590	523
	Patients’ experience	Rank of patients’ satisfaction among ten districts in Shenzhen City	Second		First	First
	Cost	Cost of all types of health care for each registered resident in the group	–		$675.3	$844.2

*CHS is short for community health station. **Time period of the data is from Jun 2014 to Dec 2015.

### Results of evaluation

*Structure evaluation.* In respect of infrastructure, the business area of community health stations increased from 410m^2^ in Jun 2015 to 903m^2^ in Jun 2017. The assets value of equipment across all community health stations increased from $2.73 million in Jun 2015 to $4.04 million in Jun 2017. The number of general practitioner doubled. The number of public health physician increased from 2 to 30, while 49 specialists set up clinics in community health stations, and 238 family doctor teams were developed during the same period.

*Process evaluation.* By June 2017, 580,000 residents had been registered with general practitioners in the hospital group. The proportion of all hospitalizations going to the group hospitals increased, which reduced the cost of health insurance in the whole hospital group. In the group. The proportion of outpatient visits in community health stations increase from 29.49% to 42.60%. This is a promising indicator that community health stations are acting for gatekeeper of the hospital group. Meanwhile, collaboration between district hospitals and community health stations promoted patient referral in the hospital group. No patient was referred from hospitals to community health stations for follow-up or rehabilitation services in 2015, but in 2016 over 10,000 patients were referred from hospitals in the group to community health stations to receive the right care at the right place.

*Outcome evaluation.* During the past two years, 4,596 more patients with diabetes, 4,995 more patients with hypertension and 822 more patients with severe mental illness were enrolled in case management (Table 1). Compared with Jun 2014–Dec 2015, there were more new cancer cases per month identified during Jun 2015–Jun 2016. There was a decrease in pneumonia cases in the second year after reform. Residents’ satisfaction with community health stations in Luohu ranked first among the ten districts of Shenzhen in 2015 and 2016. However, the mean cost per resident of all types of health care increased from $675.3 to $844.2.

## Discussion

### Stakeholders pushing the reform

The District government of Luohu gave priority to health, and set the direction for the reform by “shifting focus from treatment to health”. The District government helped the District Health and Family Planning Commission to coordinate with the Ministry of Finance, Human Resources and Social Security Bureau and Social Insurance Fund Administration, to ensure that supporting measures would be in place. Further, the District government increased financial subsidies to the group, especially community health stations. In 2016, the Ministry of Finance invested $112 million (accounting for 27.2% of all health expenditures in the district) in the group.

Staff in the group contributed efforts to the reform. Physicians and nurses adopted a philosophy of serving patients, improving treatment capacity, and strengthening collaboration with team members. Meanwhile, the reform of salary payments enhanced the enthusiasm of all staff in the group.

Along with staff in the hospital group, residents helped create and share processes and outcomes of the reform. Only by placing residents at the center of the system could the hospital group set the goal of constructing an integrated care system. Before the reform, patients regarded the community health station as the last choice for service, because they did not trust the quality of services available there. Now, 42.6% residents regard community health stations as the first contact for health care. Overall, demands of residents have been driving the reform.

### Strategies for integrated care

Some European projects have suggested that organizational integration alone is unlikely to deliver better outcomes, and that efforts must focus on clinical and service integration. Other researchers have suggested that effective care coordination can be achieved without the need for the formal integration of organizations [32,33]. Different from most international experience, most pilot programs [34,35] in China mostly began with organizational integration, which is a prerequisite for integrated care in China because public hospitals represent the vast majority of providers, the Social Insurance Fund Administration is more powerful than the Health and Family Planning Commission and hospitals are more powerful than primary healthcare institutions [36]. In this context, organizational integration led by the government is the first and best choice for changing providers’ behavior for three reasons. First, it makes health insurance reform possible. The city’s Social Insurance Fund Administration strictly controls the cost of each institute [37], and signs an annual agreement with them. A hospital or community health station has no voice in negotiating with the Social Insurance Fund Administration about the amount of funding or the payment method. It is not possible for the government to negotiate about health insurance reform with the Social Insurance Fund Administration for one institute, but it may be possible for a hospital group. Second, the concept of integrated care promotes collaboration among providers. Under the “Global budget, balance retained” policy, hospital revenue before the reform changed to cost to the hospital group after the reform. Therefore, hospitals are willing to collaborate with community health stations to reduce cost. Last but not least, integration makes full use of the power to change physicians’ behaviors. The Chinese Medical Doctor Association is responsible for the regulation and the qualification of doctors, but it cannot control or regulate the behavior of each doctor. Only institutions in the group may have an impact on physicians’ behavior by hiring, assessing and dismissing them. In summary, organizational integration provided the first step to integrated care and it provides a basis for the adoption of other strategies in China.

However, a potential disadvantage of the Luohu model is the lack of clinical integration strategies. There is no shared cross-institutional clinical pathway or guideline, both of which are tools to promote clinical integration. However, Luohu is designing its second-round of reform and taking clinical integration into account.

### Evaluation of integrated care

There are two limitations of the evaluation performed in this study. First, the evaluation was conducted two years after establishment of the group, which may be too short a time period. Rutten-van Mölken [38] suggested that some EU-funded projects may need a minimum of five years to prove themselves. On the other hand, limited comparative indicators cannot show the complete picture of reform, and the lack of individual data did not support statistical analysis of the effect. Data such as coordination among institutions and each institute’s capacity for providing services were not collected before June 2015. So, some effects cannot be measured by before-after comparison. Compared with other pilot cases, the percentage of patients who were two-way referral was higher, as was the percentage of patients going to community health stations for care [39,40]. In addition, the proportions of patients with diabetes, hypertension, and severe mental illness under case management were higher than the mean proportion (38%) in Guangdong province [41,42].

The goal of “less financial burden” was not achieved in the past two years, with more new cancer cases and excess expenditure by the health insurance fund. However, this may have resulted from the short-term increase in demand for health services as new screening programs for residents over 50 identified previously unknown cases. At the same time, prevention programs conducted in the group, such as pneumonia vaccination for residents over 60 years old, also increased cost. However, the prevention program should reduce demands for care in the future. Hence, it is necessary to study the effects of the Luohu model on financial burdens over a longer term.

According to design in the second-round reform of the Luohu model, general management center will be responsible for evaluation in the group, collaborated with information center. They will adopt more indicators related to people-centered care, population health and financial burden. It will support time series analysis of the effect in the future.

### More lessons from Luohu

The ‘one size fits all’ approach does not apply here. China’s National Health and Family Planning Commission introduced lessons from the Luohu model to all cities nationwide, but not every city could or should adopt all its strategies. Each community should design its models in the context of the local healthcare system.

In addition to the core strategies identified above, three additional points can inform policy makers. The first is to involve multiple stakeholders, especially payers, in policy making and strategy designing. In China, the Health and Family Planning Commission is responsible for the population’s health, but various ministries have the means to control the provision of health care. The Development and Reform Commission plans and sets prices for health care. The Human Resources and Social Security Bureau allocates insurance funds for health care and decides on human-resource allocations. The Ministry of Finance sets the financial budget for health. In Luohu, the District government led the reform and coordinated the joint activities of the Health and Family Planning Commission and other ministries, especially the Human Resources and Social Security Bureau. Researches [38,43] in other countries have identified the important role of health insurance in providing integrated care. We therefore suggest that the conduct of health reforms be aligned with payment reforms in health insurance. Second, normative integration could be promoted by the existence of a shared mission and work values. Normative integration is a less tangible but essential feature to promote inter-sectorial collaboration and ensure consistency between tiers of an integrated system [26]. In Shenzhen, community health stations have been affiliated with district hospitals since 2011. This has provided a shared mission, management, and values that provide a foundation for mutual trust and collaboration among them. Third, as suggested by the World Health Organization’s Global Strategy on people-centered and integrated health services [43], engagement of residents and community is essential for health reform. On the one hand, to meet their demands, residents registered with the Luohu model were involved in policy making and strategy designing in the hospital group. On the other hand, for example, the hospital group collaborated with the administrators of sports and social care to construct jogging trails for residents, promoting the benefits of exercise. The Luohu model engages not only residents but also all potential resources in the community.

## Conclusion

China’s vanguard community-based integrated care model emerged in the Luohu district as a response to the challenges of the epidemiological transition and accelerated population aging in China. Although the Luohu model was introduced to all cities in China, its effectiveness in other healthcare systems are uncertain. As the first case recognized by the National Health and Family Planning Commission, the core strategies and mechanisms of the Luohu model will promote integrated care in urban China and other countries facing the same challenges. Moreover, they will benefit universal health coverage and the realization of the full potential of the contributions of primary health care to sustainable development goals [44].
